# Electrophilic allenes participate in polar Diels–Alder reactions: study of the reactivity and site-, regio- and stereoselectivity from a molecular electron density theory perspective

**DOI:** 10.1039/d6ra02126c

**Published:** 2026-06-01

**Authors:** Alicja Bigosińska, Dominika Gondek, Luis R. Domingo, Agnieszka Kącka-Zych

**Affiliations:** a Cracow University of Technology, Faculty of Chemical Engineering and Technology, Department of Organic Chemistry and Technology Warszawska 24 31-155 Cracow Poland agnieszka.kacka-zych@pk.edu.pl; b Independent Researcher Av. Tirso de Molina 20 46015 Valencia Spain luisrdomingo@gmail.com

## Abstract

The reactivity and selectivities in the polar Diels–Alder (DA) reactions of two 1-sulfonyl allenes with two cyclic dienes have been studied within the Molecular Electron Density Theory (MEDT) at the M06-2X/6-311G(d,p) computational level. DFT-based reactivity indices characterize sulfonyl allenes as strong electrophiles and cyclic dienes as strong nucleophiles participating in polar reactions. The polar DA reaction of methylsulphonyl allene with cyclopentadiene presents a low activation enthalpy of 11.3 kcal mol^−1^, and is strongly exothermic, with a reaction enthalpy of −39.9 kcal mol^−1^. This DA reaction is completely site selective and exhibits some *endo* stereoselectivity. The polar DA reaction with 2-methoxy-cyclopentadiene presents a lower activation enthalpy of 4.1 kcal mol^−1^ and is completely *para* regioselective. The more favorable transition state structures (TSs) show an asynchronous C–C single bond formation controlled by the most electrophilic C2 carbon of the sulphonyl allenes. The analysis of the ground state of global electron density transfer (GEDT) at TSs points out the polar character of these DA reactions. A relative interacting atomic energy (RIAE) analysis reveals that while the sulfonyl allene framework is stabilized at TSs *via* GEDT, the diene framework is destabilized. The higher stabilization of the former enables the reduction in activation energies. The strong stabilization of the intra-atomic energies of the sulphur atom of the sulphonyl group is the main atomic-level electronic factor responsible for the acceleration of these polar DA reactions. The presented MEDT study will contribute to the design of further reactions involving allenes, providing deeper insight into the structure and reactivity, even through the analysis of the reagents in their ground state.

## Abstract 2

The reactivity and selectivities in the polar Diels–Alder (DA) reactions of two 1-sulfonyl allenes with two cyclic dienes have been studied within the Molecular Electron Density Theory (MEDT) at the M06-2X/6-311G(d,p) computational level.

## Graphical abstract

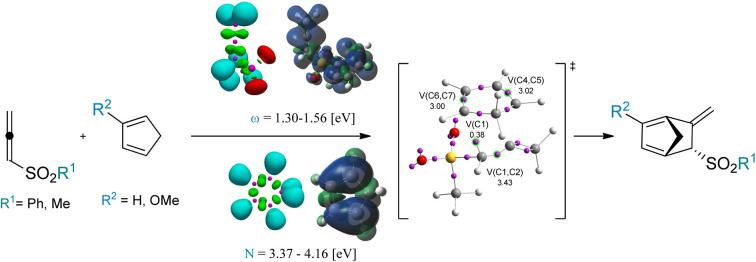

## 1. Introduction

Diels–Alder (DA) reactions,^1^ which belong to the general class of cycloaddition reactions, are among the most useful synthetic reactions in organic chemistry, as they allow the construction of six-membered carbocyclic compounds with high regio- and stereoselectivity in a single synthetic step.^2–4^ The DA reactions have been extensively studied both experimentally and theoretically.^5^ By varying the nature of the diene and ethylene, many different types of six-membered cyclic structures can be synthesized, but not all possibilities are experimentally feasible. Thus, the DA reaction between butadiene (1) and ethylene (2), selected by Hoffmann as the prototype of DA reactions,^6^ does not take place in the laboratory; it must be forced to take place after 17 hours at 165 °C and 900 atmospheres, following which it gives a 78% yield.^7^

**Scheme 1:**
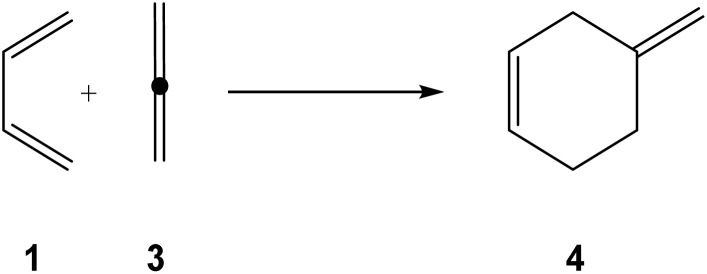
DA reaction between butadiene (1) and allene (3), yielding methylenecyclohexene (4).

The use of allene (3) as the ethylene component permits the synthesis of an exocyclic compound (3) (see Scheme 1). However, like the DA reaction of butadiene (1) with ethylene (2), it is expected that the DA reaction with the simplest allene (3) will be very unfavorable.

**Scheme 2:**
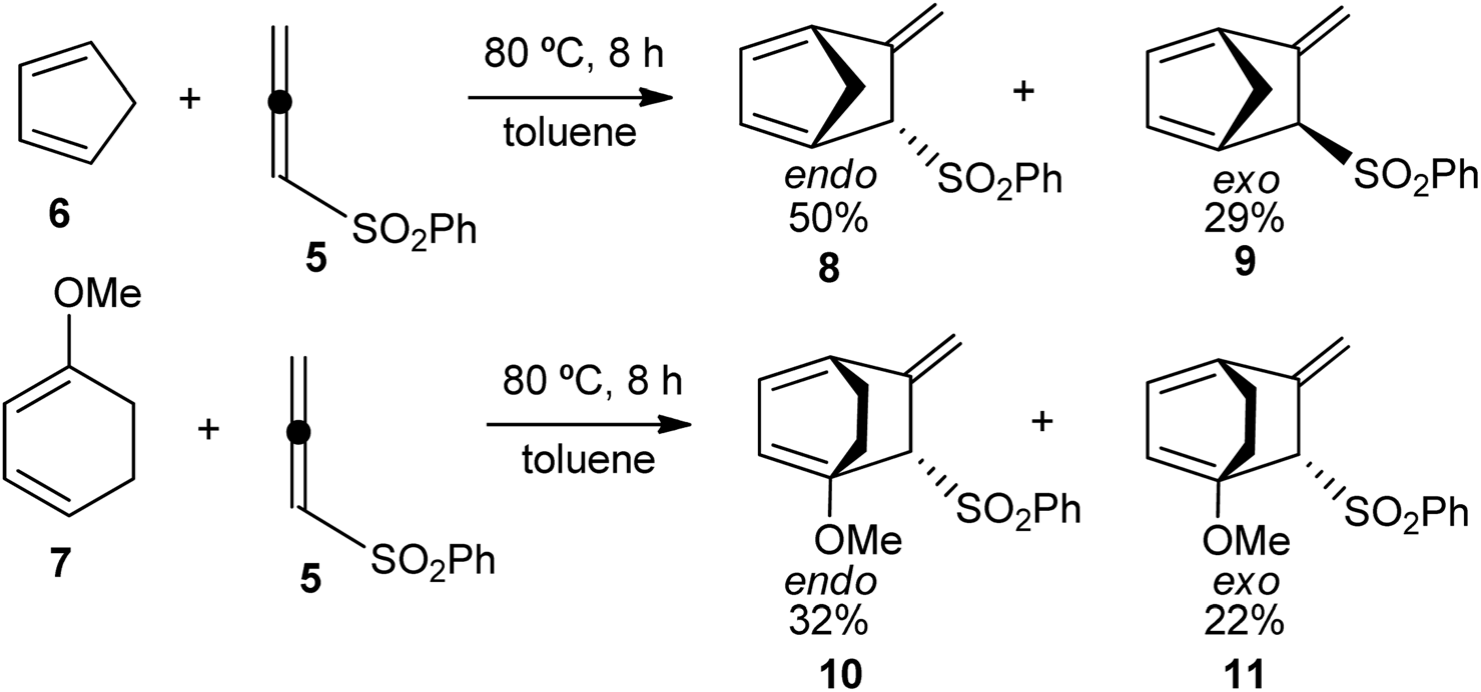
Site- and regioselective DA reactions of PhSO_2_-allene (5) with Cp (6) and Ch-OMe (7).

In 1985, Kanematsu *et al.* reported the site-selective and regioselective DA reactions of phenylsulfonyl allene (5) with cyclopentadiene (Cp) (6) and 1-metoxy-cyclohexadiene (Ch-OMe) (7).^8^ These DA reactions were completely site-selective and regioselective and exhibited low *endo* stereoselectivity (see Scheme 2). Molecular orbital calculations were used to explain, within the frontier molecular orbital^9^ (FMO) theory, the high reactivity of PhSO_2_-allene (5) and the regioselectivity in these DA reactions.

Numerous theoretical studies have been conducted on the DA reaction. An exhaustive theoretical study of experimental DA reactions allowed the establishment of the definitive role of the global electron density transfer^10^ (GEDT) occurring at the TSs in the decrease in the activation energies of the DA reactions.^11,12^ This finding enabled the establishment in 2009 of the mechanism for polar DA reactions,^13^ in which the favorable nucleophilic/electrophilic interactions taking place at the transition state structures (TSs) are responsible for the feasibility of the experimental DA reaction.^13^ Thus, the DA reactions with a low GEDT of 0.05 e were classified as non-polar DA reactions, while the DA reactions with a high GEDT of 0.20 e were classified as polar DA reactions. The analysis of the electrophilicity^14^ (*ω*) and nucleophilicity^15^ (*N*) index became a powerful quantum chemical tool to study the polar character of a DA reaction and, consequently, its experimental feasibility.

After twenty years of theoretical studies on chemical organic reactivity, Domingo proposed the Molecular Electron Density Theory^16^ (MEDT) in 2016 to study chemical organic reactivity. This theory states that the energy cost associated with the reorganization of the molecular electron density along a reaction path determines chemical reactivity. Accordingly, MEDT rejects any theoretical model based on MO analysis, such as the FMO theory^9^ and Morokuma-based energy decomposition analysis (EDA).^17–19^ MEDT has been used to investigate many organic reactions.

Very recently, an EDA based on the interacting quantum atoms^20^ (IQA), which performs a decomposition of the Kohn–Sham energies^21^ derived from DFT^22^ calculations, namely the relative interacting atomic energy^23^ (RIAE), was introduced within MEDT. RIAE enables the analysis of the atomic-level electronic interactions responsible for the activation energies of organic reactions, which constitutes the foundation of MEDT.^16^ The limitation of this approach is the partition of the molecular space into individual atomic regions based on Bader's Atoms-in-Molecules (AIM) theory.^24^ The RIAE analysis has proved to be a powerful tool in the study of the intra-atomic and interatomic interactions responsible for the activation energies of organic reactions, such as Diels–Alder (DA) reactions.^20,21,23^ The analyses of the *ξE*totalX total energies of the two interacting frameworks (*f*(*X*)) at the TSs have shown that the electronic energy stabilization of the electrophilic frameworks, resulting from the GEDT, is the cause of an effective decrease in the energies.

**Scheme 3:**
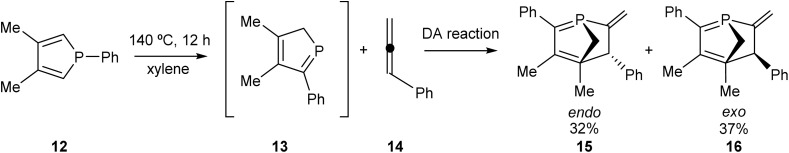
Site- and regioselective hetero DA reaction between phenylallene (14) and the generated *in situ* 2*H*-phosphole (13).

The free-metal DA reactions of linear allenes have been theoretically studied.^27–29^ The hetero DA reaction between 2H-phospholes and aryl allenes was recently experimentally studied by Tian *et al.* (see Scheme 3).^30^ The DA between phenylallene (14) and the generated *in situ* 2H-phosphole (13) was computationally studied using DFT methods at the B3LYP-D3/6-31G(d,p) computational level in xylene.^30^ The activation energy associated with the most favorable regioselective reaction path was found to be 16.3 kcal mol^−1^. The distortion/interaction EDA^19^ was performed to analyze the regioselectivity, suggesting that the most favorable regioisomeric TS had the lowest distortion energy of 15.7 kcal mol^−1^ and a similar interaction energy to those of the other TSs, indicating that the distortion energy contributed significantly to the regioselectivity.

**Fig. 1:**
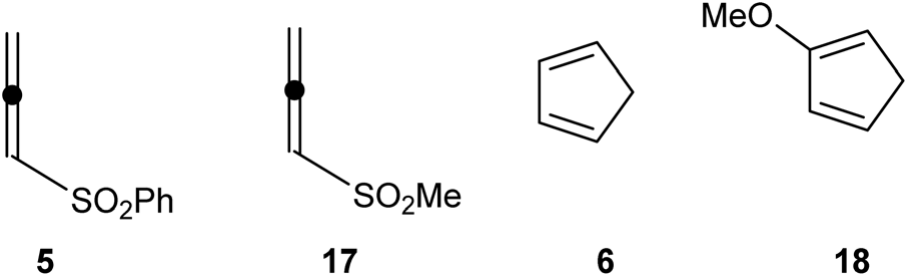
Allenes (5 and 17) and cyclic dienes (6 and 18) studied herein.

Herein, a MEDT study of the polar DA reactions of methylsulfonyl allene (17) as a model of experimental allene (5) with Cp (6) and 2-metoxycyclopentadiene (Cp-OMe) (18) is carried out in order to understand the participation of sulfonyl allenes in the DA reactions, as well as the different selectivities experimentally observed (see Fig. 1).^6^ A RIAE analysis of these polar DA reactions is carried out to determine the atomic-level electronic factors responsible for the activation energies. Reactions involving allenes are widely known experimentally,^6,7,25^ but their mechanism has not been fully elucidated. Therefore, to fill this gap, we decided to conduct research based on MEDT,^12^ in this area, as understanding the reaction mechanism will allow for the rational planning of subsequent processes.

## 2. Computational details

This MEDT study was conducted using chemical calculations within the framework of density functional theory^31^ (DFT). The M06-2X^32^ exchange-correlation functionals were employed in combination with the standard 6-311G(d,p) basis set,^33^ which incorporates d-type polarization on second-row elements, as well as p-type polarization functions on hydrogen atoms, throughout this MEDT study. TSs were verified by the presence of a single imaginary frequency. Geometry optimizations were performed using the Berny algorithm.^34,35^ The intrinsic reaction coordinate (IRC) approach^36^ was applied to confirm the unique connectivity between each TS and its associated minima.^37,38^ Solvent effects were incorporated by fully reoptimizing the gas-phase stationary points at the same theoretical level using the polarizable continuum model^39,40^ (PCM) within the self-consistent reaction field (SCRF) framework,^41–43^ with toluene as the solvent. Enthalpies, entropies, and Gibbs free energies at the M06-2X/6-311G(d,p) level in toluene were computed using standard statistical thermodynamics at 80 °C and 1 atm.^32^

GEDT values^10^ were evaluated according to the following expression: GEDT(*f*) = Σ*q*_*f*_, where *q* corresponds to the natural atomic charges^44,45^ of the atoms defining each framework (*f*) at the TS geometries. The DFT-based global reactivity indices^46,47^ were obtained using the formulations reported in ref. 45.

All quantum chemical calculations were carried out with the Gaussian 16 software package.^48^ The ELF analyses of the M06-2X/6-311G(d,p) single-determinant wavefunctions were performed using the TopMoD program^49^ on a cubic grid, with a step size of 0.1 Bohr. Molecular structures and ELF basin attractors were visualized with GaussView.^50^ ELF localization domains were represented using the ParaView software at an isovalue of 0.75 a.u.^51,52^

The IQA methodology applied in the RIAE analysis was carried out using the AIMAll software suite^53^ with M06-2X/6-311G(d,p) single-determinant pseudo-wave functions in the gas phase. It should be noted that AIMAll supports only B3LYP- and M06-2X-based wave functions in the gas phase and does not allow the inclusion of solvent effects through PCM calculations.

## 3. Results and discussion

This MEDT study was conducted in six different parts: (i) first, an electron localization function^52^ (ELF) analysis of the structures of the allenes (5 and 17) and the cyclic dienes (6, 7, and 18) was conducted; (ii) next, an analysis of the DFT-based reactivity indices^46,47^ for the reagents at the ground state (GS) was carried out. (iii) In the third part, the reaction paths associated with the DA reaction of the allenes (5 and 17) with Cp (6) were analyzed; (iv) afterward, the regioselectivity in the DA reactions of methylsulphonyl allene (17) with the substituted diene Cp-OMe (18) was studied. (v) In the fifth section, an ELF topological analysis of TSs was performed in order to characterized the bonding changes at these stationary points, and finally, (vi) a RIAE analysis of the DA reactions of the allenes (5 and 17) with Cp (6) and Cp-OMe (18) was conducted in order to establish the atomic-level electronic factors responsible for the activation energies.

### 3.1. ELF analysis of the GS structure of the reagents

**Fig. 2:**
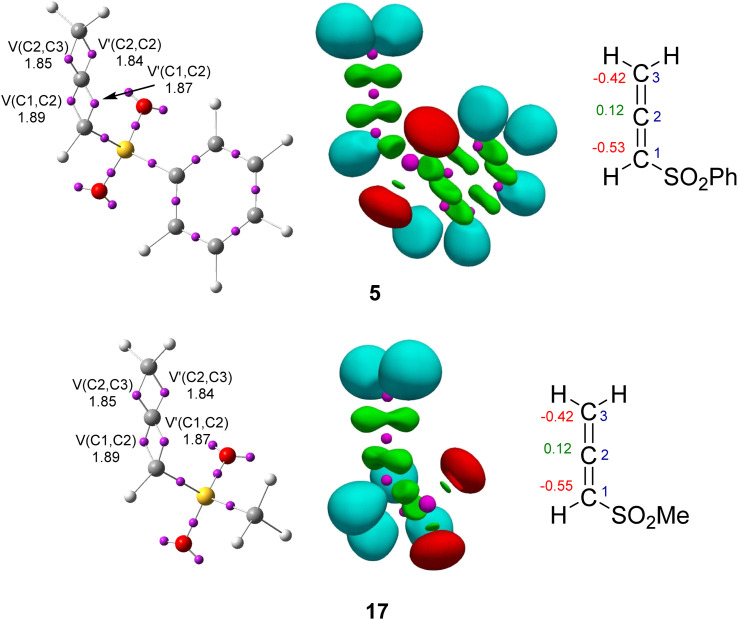
ELF valence attractor positions, along with their corresponding populations, NPA atomic charges (negative values in red and positive values in green in e), ELF valence basins, and proposed Lewis-like structures for phenylsulphonyl allene (5) and methylsulphonyl allene (17). The populations are given as the average number of electrons, e.

**Fig. 3:**
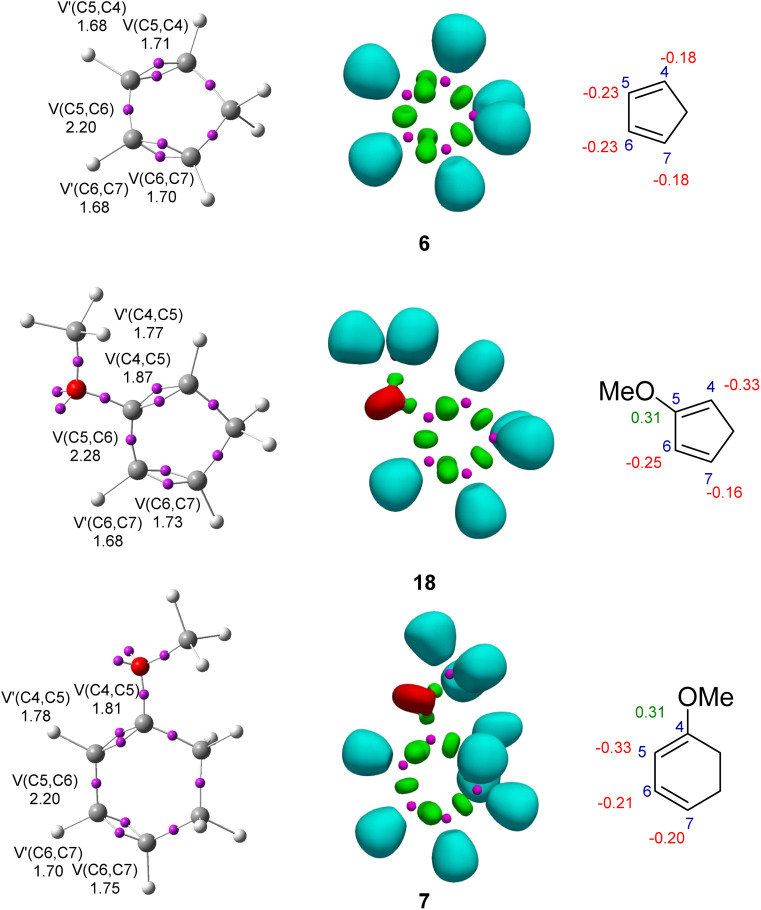
ELF valence attractor positions, along with their corresponding populations, NPA atomic charges (negative values in red and positive values in green in e), ELF valence basins, and proposed Lewis-like structures for the cyclic dienes (6, 7 and 18). The populations are given as the average number of electrons, e.

The topological analysis of the ELF^54^ at the GS allows a quantitative and qualitative description of the electronic structure of molecules.^55^ Thus, an ELF topological analysis of the electronic structure of the allenes (5 and 17) and the cyclic dienes (Cp (6), Cp-OMe (18) and CHD-OMe (7)) at the GS was performed. The position of the ELF attractors, the populations of the most relevant ELF valence basins, the natural atomic charges and the proposed Lewis-like structures for reagents are given in Fig. 2 and 3.

ELF of phenylsulphonyl allene (5) showed the presence of two pairs of disynaptic basins, *V*(C1,C2) and *V*′(C1,C2) and *V*(C2,C3) and *V*′(C2,C3), integrating a total of 3.76 e and 3.69 e, respectively. These disynaptic basins were associated with the two C1–C2 and C2–C3 double bonds of the allene moiety (Fig. 2). Methylsulphonyl allene (17) showed the same ELF topology. The populations of the disynaptic basins characterizing allene 17 were practically identical to those of allene 5. The presence of the electron-withdrawing (EW) phenyl or methylsulphonyl groups at the C1 carbon of the allene only slightly polarized the electron density of the C1=C2=C3 framework towards the C1=C2 double bond.

The ELF of Cp (6) showed the presence of two pairs of disynaptic basins, *V*(C4,C5) and *V*′(C4,C5) and *V*(C6,C7) and *V*′(C6,C7), integrating a total of 3.38 e for each pair, and one *V*(C5,C6) disynaptic basin integrating 2.20 e, characterizing the C4=C5–C6=C7 bonding region of the diene.

The ELF of Cp-OMe (18) was similar to that of Cp (6). Only the population of the *V*(V4,C5) and *V*′(V4,C5) and that of the *V*(V5,C6) disynaptic basins were slightly increased by 0.26 and 0.08 e, respectively, as a consequence of the presence of the electron-releasing (ER) OMe group at the C5 carbon (see Fig. 3).

The ELF of CHD-OMe (7) showed the presence of two pairs of disynaptic basins, *V*(C4,C5) and *V*′(C4,C5) and *V*(C6,C7) and *V*′(C6,C7), integrating a total of 3.59 and 3.45 e, respectively, which characterized the two C4–C5 and C6–C7 double bonds, and one *V*(C5,C6) disynaptic basin integrating 2.20 e, associated to the C5–C6 single bond. The presence of the ER-OMe group at C5 already slightly polarized the diene system towards the C4–C5 double bonds.

The Natural Population Analysis (NPA) of the sulphonyl allenes (5 and 17) showed that the C1 and C3 carbons were negatively charged by −0.53 (−0.55) and −0.42 e, respectively, while the central C2 carbon was positively charged by +0,12e (see Fig. 2). The NPA analysis of Cp (6) indicated that the four carbons of the diene system were negatively charged by −0.18 (C4) and −0.24 (C5) e. The presence of the ER-OMe group in Cp-OMe (18) and in CHD-OMe (7) made the substituted C4 and C5 carbons positively charged by +0.35 e due to the more electronegative character of the oxygen than the carbon atom. The other carbon atoms were negatively charged between −0.16 and −0.33 e.

### 3.2. Analysis of the global and local DFT-based reactivity indices of the reagents

In order to understand the participation of regents in polar DA reactions, an analysis of the DFT-based reactivity indices at the GS of reagents was performed.^46,47^ All reactivity indices were calculated at the B3LYP/6-31G(d) level, consistent with the theoretical definitions of the electrophilicity and nucleophilicity scales.^47^ The electronic chemical potential (µ),^56^ chemical hardness (*η*), electrophilicity (*ω*)^14^ and nucleophilicity (*N*)^15^ indices are collected in Table 1.

**Table 1:** B3LYP/6-31G(d) electronic chemical potential (*µ*), chemical hardness (*η*), electrophilicity (*ω*), and nucleophilicity (*N*) in eV for allenes (5 and 17) and cyclodienes (6, 7 and 18)

	*µ*	*η*	*ω*	*N*
5	−4.32	5.96	1.56	1.82
17	−4.18	6.72	1.30	1.58
6	−3.01	5.49	0.83	3.37
7	−2.72	5.38	0.69	3.71
18	−2.55	4.82	0.68	4.16
3	−3.30	7.71	0.70	1.97

The electronic chemical potentials (*µ*) of sulphonyl allenes (5 and 17) were −4.32 and −4.18 eV, respectively, which were positioned below those of cyclic 1,3-dienes, between −2.55 (18) and −3.01 (6) eV. This indicated that in a polar DA reaction, the GEDT^8^ will proceed from the cyclic dienes (6, 7 and 18) towards the allenes (5 and 17), as classified by forward electron density flux (FEDF).^57^

Based on the electrophilicity scale,^47^ the sulphonyl allenes (5 and 17), with *ω* = 1.82 and 1.58 eV, respectively, were classified as a strong electrophile and a moderate electrophile, respectively. Based on the nucleophilicity scale,^47^ the sulphonyl allenes (5 and 17), with *N* < 2.00 eV, were classified as marginal nucleophiles. The simplest allene (3) had electrophilicity (*ω*) and nucleophilicity (*N*) indices of 0.70 and 1.97 eV, respectively, thereby classifying it as a marginal electrophile and marginal nucleophile. Consequently, it is expected that allene 3 will not participate in the polar DA reactions.

In turn, Cp (6) was classified as a moderate electrophile with *ω* = 0.83 eV and a strong nucleophile with *N* = 3.37 eV. The presence of the ER-OMe group on the cyclic dienes (7 and 18) decreased their electrophilicity (*ω*) indices to 0.60 and 0.68 eV, respectively, thereby classifying them as marginal electrophiles, and increased their nucleophilicity (*N*) indices to 3.71 and 4.16 eV, thereby classifying them as strong nucleophiles.

The analysis of the global DFT-based reactivity indices indicated that in a polar DA reaction, the allenes (5 and 17) will play the role of electrophiles, while the cyclic dienes (6, 7 and 18) will act as nucleophiles.

**Fig. 4:**
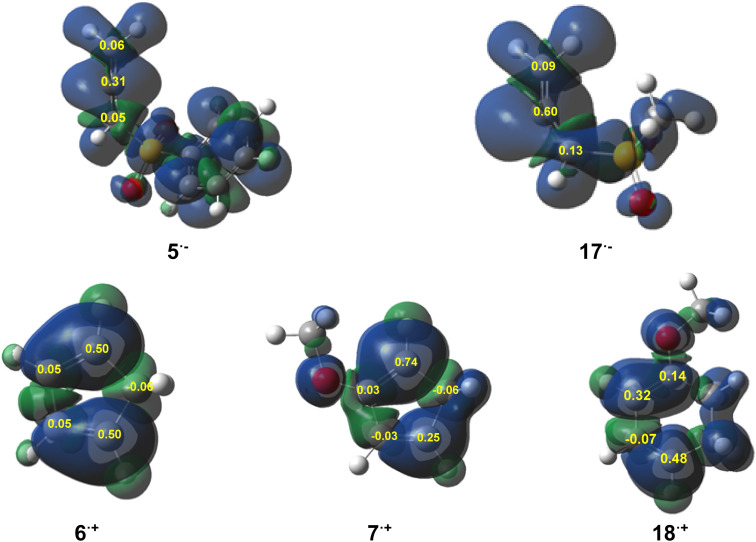
3D representations of the ASD of the radical anion (**5˙**^**−**^ and 17**˙**^**−**^) and the radical cation (**6˙**^**+**^, **7˙**^**+**^ and **18˙**^**+**^), together with the electrophilic (P_k_^+^) Parr functions of 5 and 17 and the nucleophilic (P_k_^−^) Parr functions of 6, 7 and 18.

Next, the electrophilic (P_k_^+^) and nucleophilic (P_k_^−^) Parr functions^58^ were analyzed to get insight into local reactivity. The electrophilic (P_k_^+^) Parr functions of allenes 5 and 17 and the nucleophilic (P_k_^−^) Parr functions of cyclic dienes 6, 7 and 18 are given in Fig. 4, while all Parr functions of the reagents are listed in Table S1 in the SI.

An analysis of the electrophilic (P_k_^+^) Parr functions of allenes 5 and 17 indicated that the central carbon C2 atom was the most electrophilic center, with P_k_^+^ = 0.31 e and 0.60 e, respectively, while the C1 and C3 carbon atoms exhibited electrophilic activation in the range of 0.05–0.13 e (see Fig. 4 and Table 2).

**Table 2:** M06-2X/6-311G(d,p) gas-phase intra-atomic (*ξE*^X^_intra_), interatomic (*ξE*^X^_inter_) and total (*ξE*^X^_total_) energies, in kcal mol^−1^, of the allene and diene frameworks at the TSs relative to their reagents in GS. The sum of the *ξE*^X^_total_ values of both frameworks, denoted as *ξE*^all+dien^_total_, represents the RIAE relative energies of these DA reactions

	*f*(*X*)	*ξE* ^X^_intra_	*ξE* ^X^_inter_	*ξE* ^X^_total_	*ξE* ^all+dien^_total_
TS4	Allene	11.8	−4.6	7.1	18.5
	Diene	35.4	−24.0	11.3	
TS1-n	Allene	−20.9	5.8	−15.0	8.8
	Diene	41.2	−17.4	23.8	
TS2-n	Allene	−13.1	−0.6	−13.7	8.9
	Diene	39.6	−17.1	22.5	
TS3-pn	Allene	−51.6	27.6	−24.0	1.0
	Diene	58.9	−33.9	25.0	

The C4 and C7 carbon of Cp (6), with P_k_^−^ = 0.50, were the most nucleophilic centers of this diene, while the C5 and C6 carbons were negligibly activated. The presence of the ER-OMe group on the C5 and C4 carbons of Cp-OMe (18) and Ch-OMe (7) markedly nucleophilically activated the C4 and C7 carbons, P_k_^−^ = 0.74 and 0.48, respectively, while the C7 carbon of (7) and the C5 carbon of (18) were activated at 0.25 and 0.32, respectively.

Based on the analysis of the local Parr functions of the reagents, the most favorable reaction path of these DA reactions will be initialized by the nucleophilic attack of the C4 carbon of Cp (6) and Cp-OMe (18) or the C7 carbon of Ch-OMe (6) on the central C2 carbon of the allenes (5 and 17).

### 3.3. Study of the DA reactions of sulphonyl allenes (5 and 17) with Cp (6)

**Scheme 4:**
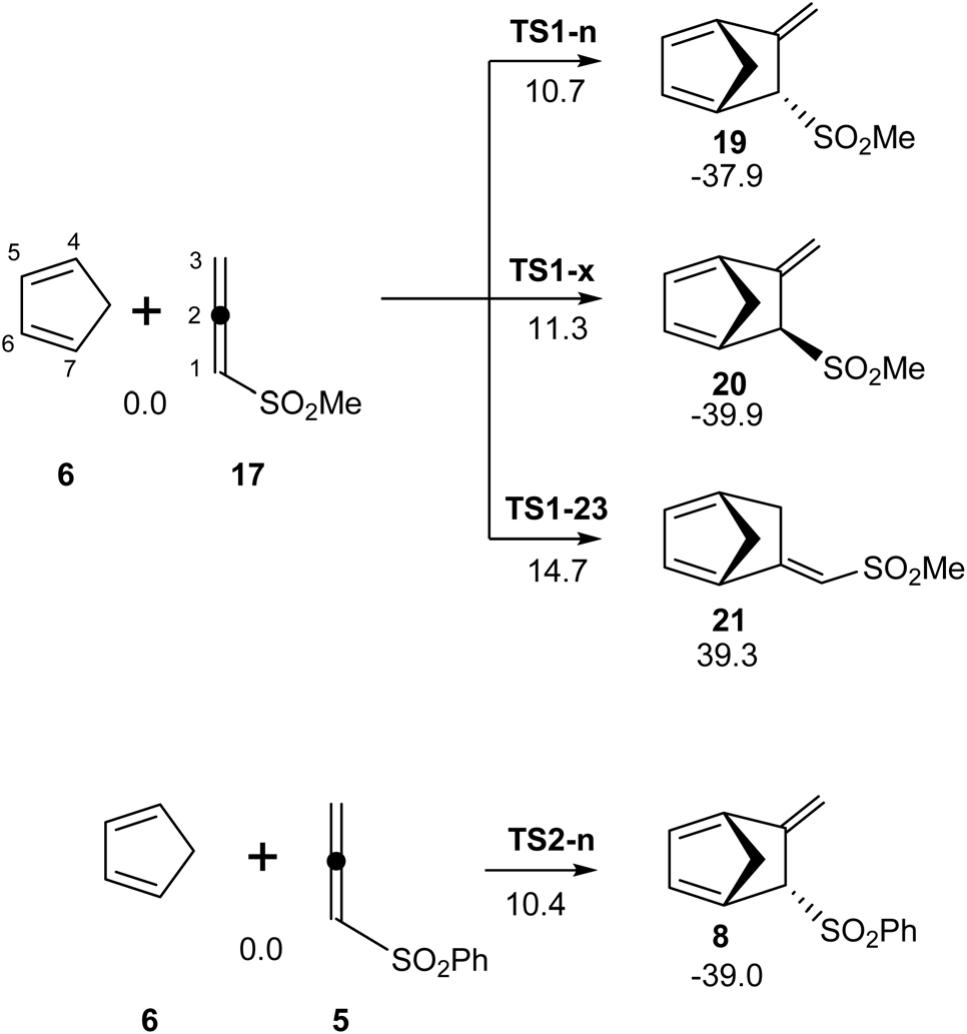
DA reactions of the allenes (5 and 17) with Cp (6). Relative enthalpies in toluene are given in kcal mol^−1^.

First, the DA reaction of sulphonyl allenes (5 and 17) with Cp (6) was studied in toluene. Due to the presence of two C–C double bonds in the sulphonyl allenes (5 and 17), three competitive reaction paths were feasible (see Scheme 4). They were associated with the stereoisomeric *endo* and *exo* approach modes of the sulphonyl group of the allenes (5 and 17) over the diene system of Cp (6) and the site isomeric approach mode of the non-substituted C2–C3 double bond of the allenes (5 and 17) to the diene system of Cp (6). The three reaction paths for the reactions involving methylsulphonyl allene (3) were studied. On the other hand, the *endo* stereoisomeric reaction path associated with the experimental DA reaction of phenylsulphonyl allene (5) with Cp (6), yielding the cycloadduct (8), was also studied (see Schemes 2 and 4). The analysis of the stationary point found along the studied reaction paths allowed us to find one TS and one cycloadduct, indicating that these polar DA reactions took place *via* a one-step mechanism. The polar DA reaction between the simple allene (3) and Cp (6) was also studied (see Scheme S1 in the SI). Relative enthalpies are given in Scheme 4, while the thermodynamic data are given in Table S2 in the SI.

Some appealing conclusions were obtained from the analysis of the relative enthalpies, as given in Scheme 4: (i) the activation enthalpies associated with the DA reaction of methylsulphonyl allene (17) with Cp (6) ranged from 10.7 to 14.7 kcal mol^−1^, the reaction being strongly exothermic in the range from −34.8 to −39.9 kcal mol^−1^ and, consequently, irreversible. Therefore, this DA reaction was under kinetic control; (ii) the activation enthalpy associated with this DA reaction *via* the most favorable TS1-n, 10.7 kcal mol^−1^, was 9.1 kcal mol^−1^ less than that associated with the non-polar DA reaction of simplest allene (3) *via*TS4, 19.8 kcal mol^−1^ (see Scheme S1 in the SI). (iii) This reaction exhibited some *endo* stereoselectivity, with *exo*TS1-x positioned 0.6 kcal mol^−1^ above *endo*TS1-n; (iv) this reaction was completely C1–C2 site-selective, with TS1-23 positioned 4.0 kcal mol^−1^ above TS1-x. Finally, (v) the activation enthalpy associated with the polar DA reaction of phenylsulphonyl allene (5) with Cp (6) *via*TS2-n was only 0.3 kcal mol^−1^ lower than that involving methylsulphonyl allene (17), while the reaction was 1.1 kcal mol^−1^ more exothermic. Consequently, the substitution of the Ph group of the experimental phenylsulphonyl allene (5) for the smaller Me group in methylsulphonyl allene (17) did not cause any significant energy change.

**Fig. 5:**
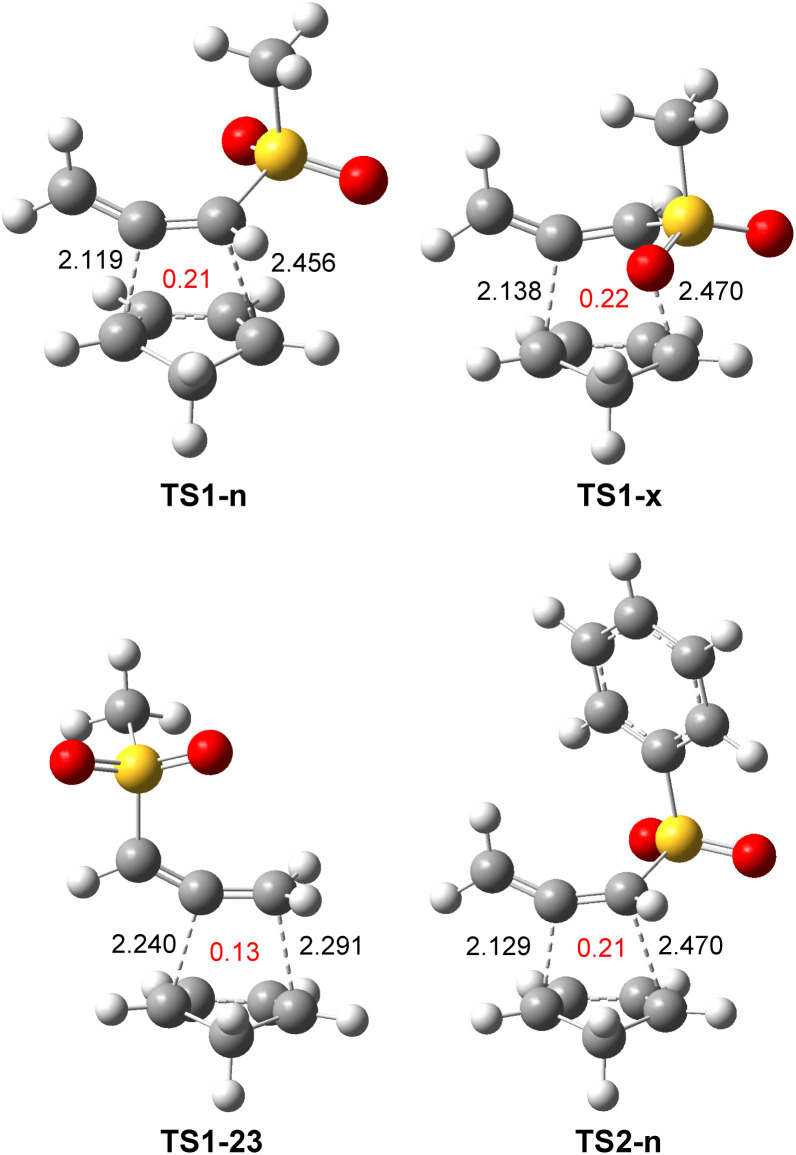
M06-2x/6-311G(d,p) optimized geometries in toluene of the TSs involved in the DA reactions of the sulphonyl allenes (5) and (17) with Cp (6). The GEDT values, in red, are given as the average number of electrons, e.

The optimized geometries of TSs involved in the polar DA reaction of the sulphonyl allenes (5 and 17) with Cp (6) are given in Fig. 5. The distance between the two pairs of interacting carbons was found in the range from 2.12 to 2.47 Å. While TSs involving the participation of the C1–C2 double bond of methylsulphonyl allene (17) were asynchronous with a Δ*l* = 0.34, that associated with the participation of the C2–C3 double bond showed a synchronous C–C single bond formation. The geometry of TS2-n involved in the polar DA reaction of phenylsulphonyl allene (5) with Cp (6) was closer to that of TS1-n. All C–C distances, which were higher than 2.0 Å, indicated that the formation of the new C–C single bonds did not begin at any of the three TSs.^10^

Finally, the GEDT at the TSs was evaluated in order to characterize the polar character of these DA reactions. GEDT values lower than 0.05 e correspond to non-polar processes, while values higher than 0.20 e correspond to highly polar processes. The GEDT values at the TSs are given in Fig. 5. The GEDT at TS2, associated with the DA reaction of the simplest allene (3) with Cp (6), presented a very low value, 0.07 e (see Fig. S1 in the SI). This low value indicated the non-polar character of this DA reaction, categorized as the null electron density flux (NEDF).^57^

The GEDT values at TS1-n and TS1-x, 0.21 and 0.22 e, respectively, illustrated the polar character of these DA reactions. This behavior was a consequence of the strong electrophilic character of methylsulphonyl allene (17) and the strong nucleophilic character of Cp (6). The flux of the electron density, which went from the Cp framework to the allene framework, categorized this polar DA reaction as FEDF, in agreement with the previous analysis of the DFT-based reactivity indices. The GEDT value at TS1-23, 0.13 e, was lower than that at TS1-n and TS1-x, as a consequence of the non-direct participation of the EW sulphonyl group in the process. Like the other reaction paths, this reaction was categorized as FEDF.

The GEDT value at TS2-n, 0.21 e, was identical to that found at TS1-n, which had a closely related activation enthalpy and geometry.

### 3.4. Study of the regioselectivity in the polar DA reactions of methylsulphonyl allene (17) with non-symmetrically substituted dienes

**Scheme 5:**
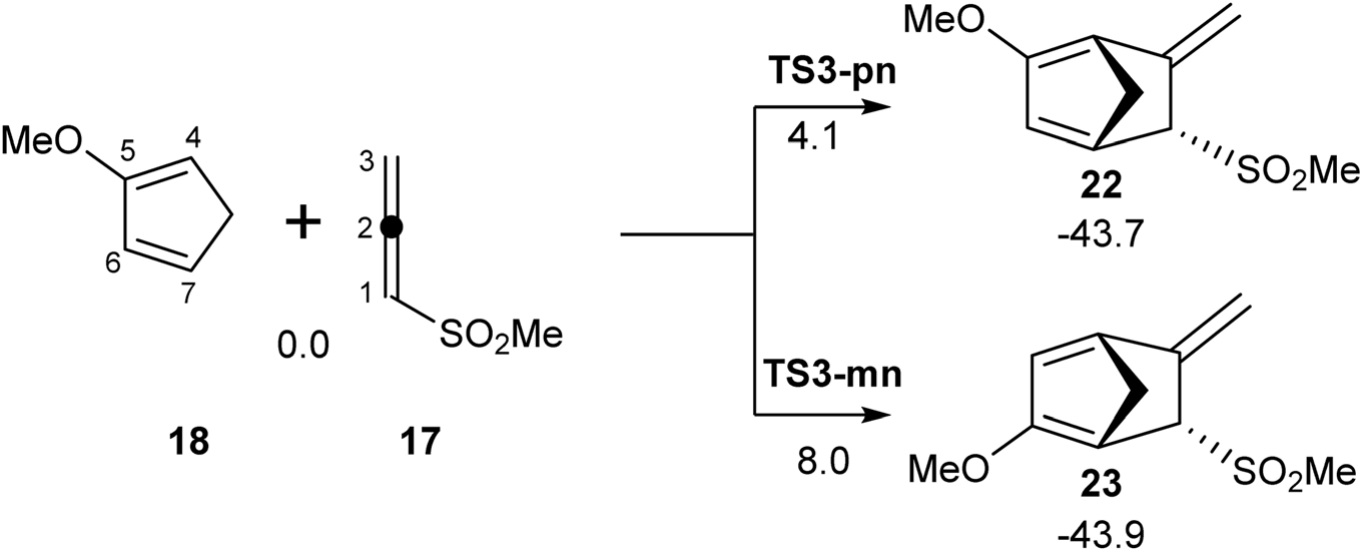
*Endo*/*para* and *endo*/*meta* regioisomeric reaction paths associated with the DA reaction between methylsulphonyl allene (17) and Cp-OMe (18). Relative enthalpies in toluene are given in kcal mol^−1^.

In the polar DA reactions of phenylsulphonyl allene (5) with 1- or 2-substituted dienes, two competitive regioisomeric reaction paths were feasible. Experimentally, only one regioisomeric cycloadduct was obtained. In order to explain the regioselectivity in these polar DA reactions, the *para/endo* and *meta/endo* regioisomeric reaction paths associated with the DA reaction between methylsulphonyl allene (17) and Cp-OMe (18) were analyzed (see Scheme 5). The relative enthalpies associated with the stationary points are given in Scheme 5, while the thermodynamic data are given in Table S2 in the SI.

The activation enthalpies associated with the two regioisomeric reaction paths were 4.1 (TS3-pn) and 8.0 (TS3-mn) kcal mol^−1^, with the reactions being exergonic by −43 kcal mol^−1^. Two appealing conclusions could be obtained from these relative enthalpies: (i) the polar DA reaction between methylsulphonyl allene (17) and Cp-OMe (18) was completely *para* regioselective, with *meta*TS3-mn positioned 3.9 kcal mol^−1^ above *para*TS3-pn, in agreement with the experimental outcomes;^8^ (ii) the activation enthalpy of the polar DA reaction between methylsulphonyl allene (17) and Cp-OMe (18) *via*TS3-pn was 6.6 kcal mol^−1^ lower than that associated with the DA reaction between this allene and Cp (6) *via*TS1-n as a consequence of the higher nucleophilic character of Cp-OMe (18) than that of Cp (6) (see Table 1).

**Fig. 6:**
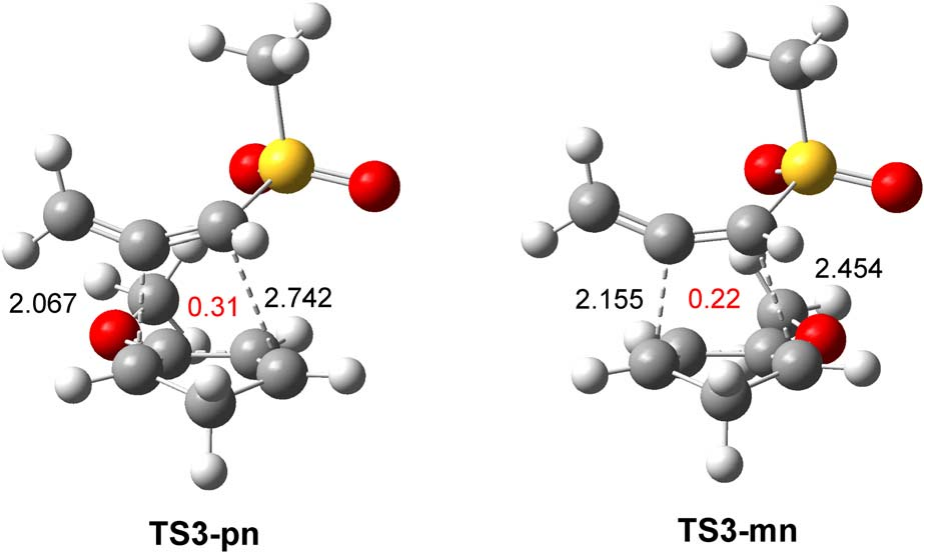
M06-2x/6-311G(d,p) optimized geometries in toluene of the regioisomeric TS3-pn and TS3-mn involved in the DA reaction of MeSO_2_-allenes (17) with Cp-OMe (18). The GEDT values, in red, are given as the average number of electrons, e.

The optimized geometries of TS3-pn and TS3-mn are given in Fig. 6. The distances between the two pairs of interacting carbons were found in the range from 2.07 to 2.74 Å. The more favorable *para*TS3-pn was more advanced and more asynchronous, Δ*l* = 0.68, than *meta*TS3-mn, Δ*l* = 0.30. All C–C distances, which were higher than 2.0 Å, indicated that the formation of the new C–C single bonds did not begin at any of the two regioisomeric TSs.^10^

The GEDT values at TS3-pn and TS3-mn, 0.31 and 0.22 e, respectively, indicated the high polar character of this DA reaction. The more favorable *para*TS3-pn was more polar than *meta*TS3-mn as a consequence of the more favorable nucleophilic/electrophilic interactions taking place at the TS along the *meta* approach mode. On the other hand, TS3-pn was more polar than TS1-n as a consequence of the more nucleophilic character of Cp-OMe (18) than Cp (6) (see Table 1). Like in the other DA reactions involving Cp (3), the flux of the electron density, which went from the nucleophilic Cp framework to the electrophilic allene one, categorized this polar DA reaction as FEDF, in agreement with the analysis of the DFT-based reactivity indices.

**Fig. 7:**
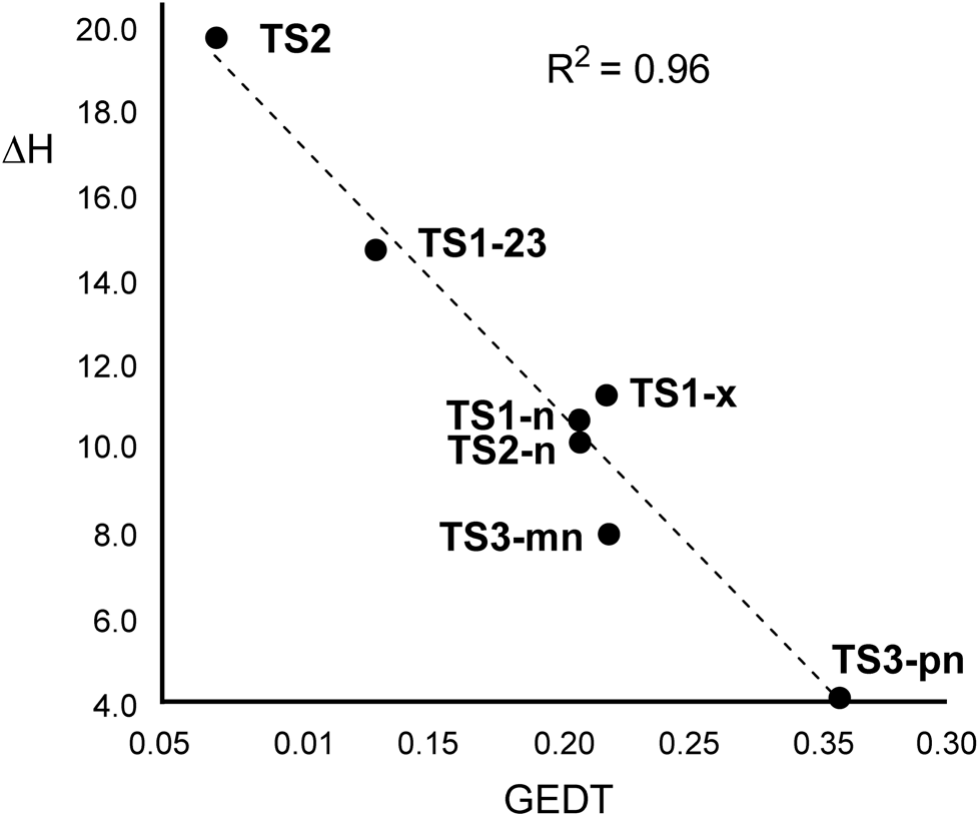
Plot of the activation enthalpies, Δ*H* in kcal mol^−1^, associated with the DA reactions of allenes (3, 5 and 17) with nucleophilic dienes *versus* the GEDT, given as the average number of electrons, e, at the corresponding TS.

Many MEDT studies on polar DA reactions have shown that the activation energies primarily depend on the GEDT taking place at the TSs.^54–62^Fig. 7 shows a graphical representation of the activation enthalpies of the DA reactions of the allenes (3, 5 and 17) studied here with respect to the GEDT taking place at the corresponding TSs. As can be seen, a very good lineal correlation was found, with *R*^2^ = 0.96. While on the upper left side is located the non-polar DA reaction between the simplest allene (3) and Cp (6), on the lower right side, a stronger polar DA reaction between the electrophilic methylsulphonyl allene (17) and nucleophilic Cp-OMe (18) is found. The deviation of the linearity may be due to additional secondary attractive or repulsive electronic effects present at the TS geometries. Thus, TS1-n and TS1-x are a pair of stereoisomers whose relative energies differ by only *ca.* 0.6 kcal mol^−1^ (see Scheme 4). While Fig. 7 confirms that activation enthalpies of *ca.* 11 kcal mol^−1^ strongly depend on the GEDT, the subtle electronic factors governing stereoselectivity are also embedded in these relative enthalpies.

### 3.5. ELF topological analysis of the TSs involved in the polar DA reactions of the sulphonyl allenes (5 and 17) with the cyclic dienes (6 and 18)

Subsequently, the electronic structures of the TSs associated with the polar DA reactions of the sulphonyl allenes (5 and 17) with the cyclic allenes Cp (6) and Cp-OMe (18), given in Schemes 4 and 5, were investigated by performing an ELF topological analysis.

#### 3.5.1. ELF topological analysis of the TSs involved in the polar DA reactions of the sulphonyl allenes (5 and 17) with Cp (6)

**Fig. 8:**
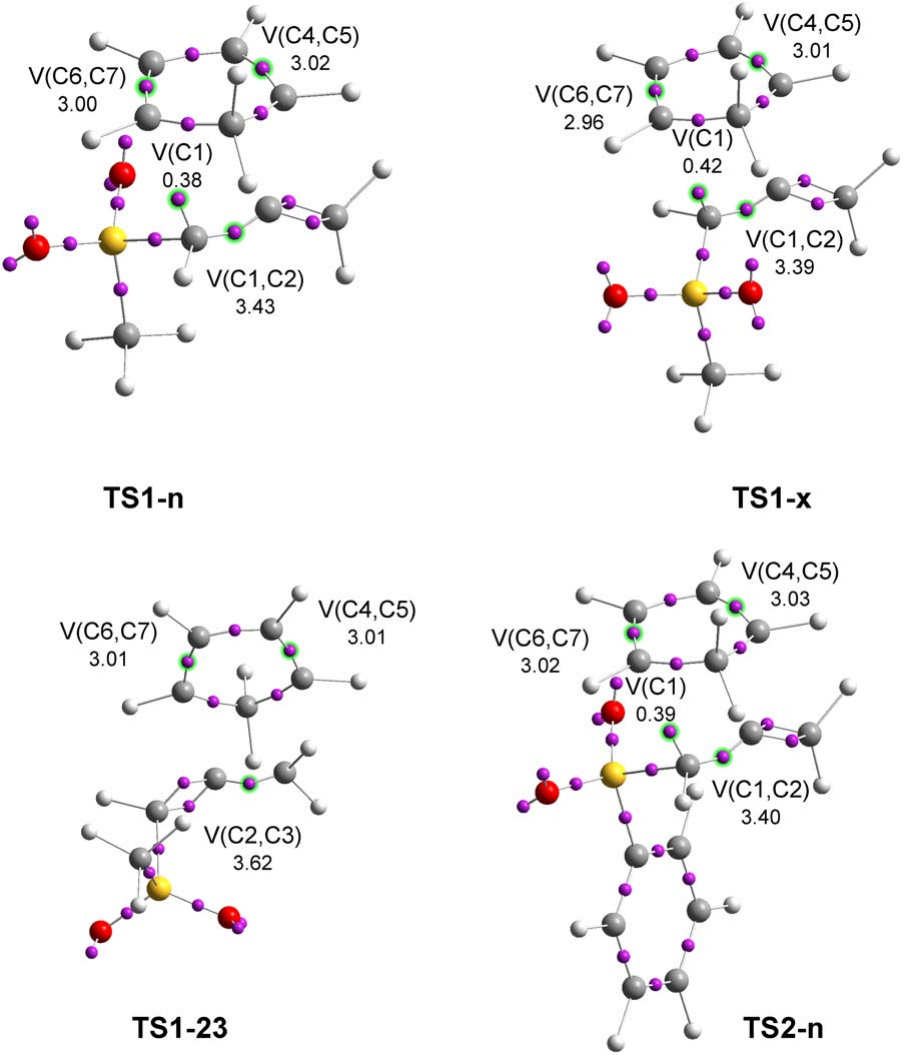
ELF basin attractor positions, along with the most relevant valence basin populations of TSs associated with the DA reactions between sulphonyl allenes (5 and 17) with Cp (6). The electron populations are expressed as the average number of electrons, e.

First, the electronic structures of the TSs involved in the polar DA reactions of the sulphonyl allenes (5 and 17) with Cp (6), given in Scheme 4, were investigated. The attractor positions of the valence basins together with the populations of the more relevant valence basins of the TSs are shown in Fig. 8.

ELF of TS1-n showed the presence of three disynaptic basins, *V*(C1,C2), *V*(C4,C5) and *V*(C6,C7), integrating 3.43 e, 3.02 e and 3.00 e, associated with the C1–C2, C4–C5 and C6–C7 bonding regions, respectively. The most relevant feature of the ELF of TS1-n was the presence of a new *V*(C1) monosynaptic basin integrating 0.38 e, which was associated with the creation of a *pseudoradical* C1 center. This *pseudoradical* center will be demanded for the subsequent creation of the new C1–C7 single bond.^8^ Non-*pseudoradical* centers at the C2, C4 and C7 carbons, which will be demanded for the creation of the new C1–C7 and C2–C4 single bonds, were observed, indicating the earlier nature of this TS.

ELF of *exo*TS1-x was very similar to that of stereoisomeric *endo*TS1-n. At TS1-x, only the population of the *pseudoradical* C1 center was slightly higher, 0.41 e higher than that present at TS1-n. The C1–C2, C4–C5 and C6–C7 bonding regions were characterized by the presence of three disynaptic basins, V(C1,C2), V(C4,C5) and V(C6,C7) basins, integrating 3.39 e, 3.01 e and 2.96 e, respectively.

ELF of site isomeric TS1-23 was characterized by three *V*(C2,C3), *V*(C4,C5) and *V*(C6,C7) disynaptic basins, which integrated 3.61, 3.01 and 3.01 e, respectively. Non-V(C) monosynaptic basins were observed at this TS, indicating its earlier nature.

The ELF of TS2-n exhibited identical behaviors to those of TS1-n, indicating that the substitution of the Ph group in the experimental phenylsulphonyl allene (5) for a Me group in the model methylsulphonyl allene (17) did not appreciably affect the electronic structure of these TSs.

Some conclusions can be obtained from the ELF analysis of the four TSs: (i) the four TSs showed a great electronic similarity. Only stereoisomeric TS1-n and TS1-x showed the presence of a new V(C1) monosynaptic basin, indicating that they were slightly more advanced than TS1-23. (ii) The C1–C2, C2–C3, C4–C5 and C6–C7 bonding regions at methylsulphonyl allene (17) and Cp (6), which were characterized by the presence of two *V*(C*x*,C*y*) and *V*′(C*x*,C*y*) disynaptic basins at the GS, were merged into only one *V*(C*x*,C*y*) disynaptic basin as a consequence of the depopulation of the C1–C2, C2–C3, C4–C5 and C6–C7 bonding regions at the TSs. These depopulations were required for the formation of the four *V*(C*x*) and *V*(C*y*) monosynaptic basins in a subsequent step of the reaction; finally, (iii) the absence of neither V(C1,C7) nor V(C2,C4) disynaptic basins indicated that the formation of the new C1–C7 and C2–C4 single bonds did not begin at these TSs.

#### 3.5.2. ELF topological analysis of the para and meta regioisomeric TSs involved in the DA reaction between methylsulphonyl allene (17) and Cp-OMe (18)

**Fig. 9:**
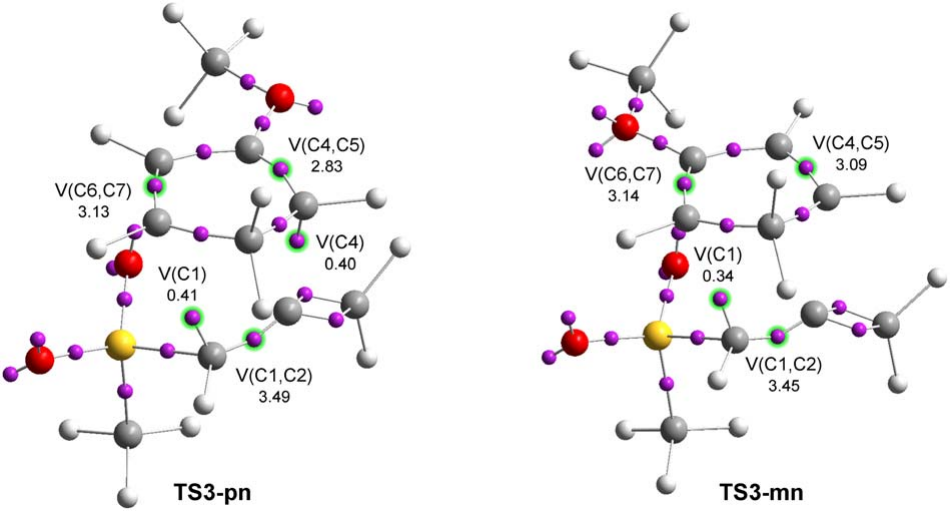
ELF basin attractor positions, along with the most relevant valence basin populations of TSs associated with the DA reaction between MeSO_2_-allene (17) and Cp-OMe (18). The electron populations are expressed as the average number of electrons, e.

Next, the electronic structures of the *para* and *meta* regioisomeric TSs involved in the polar DA reaction between methylsulphonyl allene (17) and Cp-OMe (18) were analyzed through an ELF topological analysis of the electron density (see Scheme 5). The attractor positions of the valence basins together with the populations of the more relevant valence basins of the TSs involved in the analysed reaction are shown in Fig. 9.

ELF of *para*TS3-pn showed the presence of three disynaptic basins, V(C1,C2), V(C4,C5) and V(C6,C7), integrating 3.49, 2.83 and 3.13 e and characterizing the C1–C2, C4–C5 and C6–C7 bonding regions, respectively, and two V(C1) and V(C4) monosynaptic basins, integrating 0.41 e and 0.40 e, associated with the creation of two C1 and C4 *pseudoradical* centers, respectively. On the other hand, ELF of the regioisomeric *meta*TS3-mn showed a similar electron density distribution to that present at TS3-pn. TS3-mn showed the creation of only one V(C1) monosynaptic basin, indicating its earlier nature than the more favorable TS3-pn (Fig. 9). The bonding changes found in these TSs were very similar to those found in the other four TSs. Only the most favorable TS3-pn was the most advanced TS to show the presence of two *pseudoradical* centers.

The formation of a new Cx−Cy single bond occurs by the coupling of two Cx and Cy *pseudoradical* centers created at the beginning of the reaction by the depopulation of the corresponding C−Cx[y] double bonds.^10^ Thus, these features explained the ELF topological analysis of the C1–C2, C4–C5 and C6–C7 bonding regions, which were characterized by the presence of only one *V*(C*x*,C*y*) disynaptic basin at the six TSs. This was a consequence of the depopulation of the C*x*–C*y* double bonds present at the GS of the reagents, which were characterized by the presence of two *V*(C*x*,C*y*) and *V*′(C*x*,C*y*) disynaptic basins (see Fig. 1 and 2).

### 3.6. RIAE analysis of the polar DA reactions of the sulphonyl allenes (5 and 17) with the nucleophilic dienes (Cp (6) and Cp-OMe (18))

Finally, to assess the atomic-level electronic effects of the substitution on the activation energies of the polar DA reactions of the sulphonyl allenes 5 and 17 with nucleophilic cyclic Cp (6) and Cp-OMe (18), an RIAE analysis^23^ of the more favorable TSs involved in these DA reactions was performed. The RIAE analysis offers insight into rationalizing the energy costs at the atomic level associated with electron density changes at the TSs, on which MEDT is founded.^16^ The RIAE theoretical background is outlined in ref. 23 and 60. As a reference, the non-polar DA reaction of the simplest allene (7) with Cp (6) *via*TS4 was also considered. The RIAE study was carried out at the M06-2X/6-311G(d,p) computational level in the gas phase, as required by the IQA^20^ calculations. The gas-phase intra-atomic (*ξE*^X^_intra_), interatomic (*ξE*^X^_inter_) and total (*ξE*^X^_total_) energies for the allene and the diene frameworks of the three TSs are presented in Table 2. The sum of the *ξE*^X^_total_ values of both frameworks, denoted as *ξE*^all+dien^_total_, represents the RIAE relative energies.

The RIAE analysis of TS4, corresponding to the non-polar DA reaction of the simplest allene 3 with Cp 6, revealed that both unfavorable total energies (*ξE*^all^_total_ and *ξE*^dien^_total_) associated with the allene and diene frameworks, 7.1 and 11.3 e, respectively, were responsible for the high RIAE activation energy associated with this unfavorable non-polar DA reaction.^63^ The destabilization of the diene framework contributed to a major increase in the RIAE activation energy. At TS4, while the interatomic energies (*ξE*^X^_inter_) were stabilizing by −4.6 and −24.0 kcal mol^−1^, the intra-atomic energies (*ξE*^X^_intra_) were strongly destabilizing by 11.8 and 35.5 kcal mol^−1^. This RIAE analysis was similar to that found in the non-polar DA reaction between butadiene and ethylene, in which the destabilization of both frameworks was responsible for the high RIAE activation energy, 21.26 kcal mol^−1^, of this unfavorable DA reaction.^63^

A detailed analysis of the factors responsible for the unfavorable total energies (*ξE*^X^_total_) indicated that the interatomic energies (*ξE*^C2^_inter_), 9.6 kcal mol^−1^, associated with the allene central C2 carbon, and the intra-atomic energies (*ξE*^X^_intra_) associated with the terminal C4 and C7 carbon of Cp (3), 11.9 and 9.5 kcal mol^−1^, respectively, were the main atomic-level electronic factors responsible for the high RIAE activation energies of this non-polar DA reaction.

The RIAE analysis of TS1-n, corresponding to the polar DA reaction of methylsulphonyl allene (16) with Cp (5), revealed that while the total energies (*E*^dien^_total_) associated with the diene framework was strongly destabilizing by 23.8 kcal mol^−1^, that of the allene framework was stabilizing, *ξE*^all^_total_ = −15.0 kcal mol^−1^. As a result, the RIAE activation energy of this polar DA reaction decreased to 8.8 kcal mol^−1^. Thus, while the total energies (*E*^dien^_total_) of this polar reactions were more unfavorable than those at non-polar TS2, the strong total energies (*ξE*^all^_total_) compensated this destabilization, reducing the RIAE activation energy of this polar process.

The RIAE analysis of TS2-n, corresponding to the experimental polar DA reaction of phenylsulphonyl allene (7) with Cp (6), revealed a great similarity with that of TS1-2 (see Table 2). The main difference was found in the factors contributing to the total energies (*ξE*^all^_total_) of the allene framework. While the intra-atomic energies (*ξE*^all^_intra_) were 7.8 kcal mol^−1^ less stabilizing than those at TS1-n, the interatomic energies (*ξE*^all^_inter_) were 6.4 kcal mol^−1^ more stabilizing. Despite these variations, the total energies (*ξE*^all^_total_) only varied by 1.3 kcal mol^−1^.

The RIAE analysis of high-polar TS3-pn, corresponding to the polar DA reaction of the methylsulphonyl allene (17) with Cp-OMe (18), showed the same trend as that observed at TS1-n. Interestingly, while the unfavorable total energies (*E*^dien^_total_) were more destabilizing by 25.0 kcal mol^−1^, the *ξE*^all^_total_ ones were more stabilizing by −24.0 kcal mol^−1^, thereby considerably decreasing the RIAE activation energy of this polar DA reaction to 1.0 kcal mol^−1^.

A detailed analysis of the factors responsible for the stabilizing total energies (*E*^all^_total_) total energies associated with polar TS1-n and TS2-pn revealed that while the interatomic energies (*ξE*^all^_inter_) were destabilizing by 5.8 and 27.6 kcal mol^−1^, respectively, the intra-atomic energies (*ξE*^all^_intra_) were strongly stabilizing by −20.0 and −51.6 kcal mol^−1^, respectively.

A more detailed analysis of the factors responsible for the strong stabilizing intra-atomic energies (*ξE*^all^_intra_) at the atomic-level at polar TS1-n and TS2-pn revealed that the sulfur atom of the methylsulphonyl group was the key contributor. This strong stabilization of the intra-atomic energies (*E*^S^_intra_), −30.9 and −57.6 kcal mol^−1^, respectively, resulting from GEDT taking place at these polar TSs, was the main atomic-level electronic factor responsible for the decrease in the RIAE activation energies in these polar DA reactions. Note that at TS2-n, the intra-atomic energy (*E*^S^_intra_) associated with the sulfur atom of the phenylsulphonyl group was also −25.1 kcal mol^−1^.

**Fig. 10:**
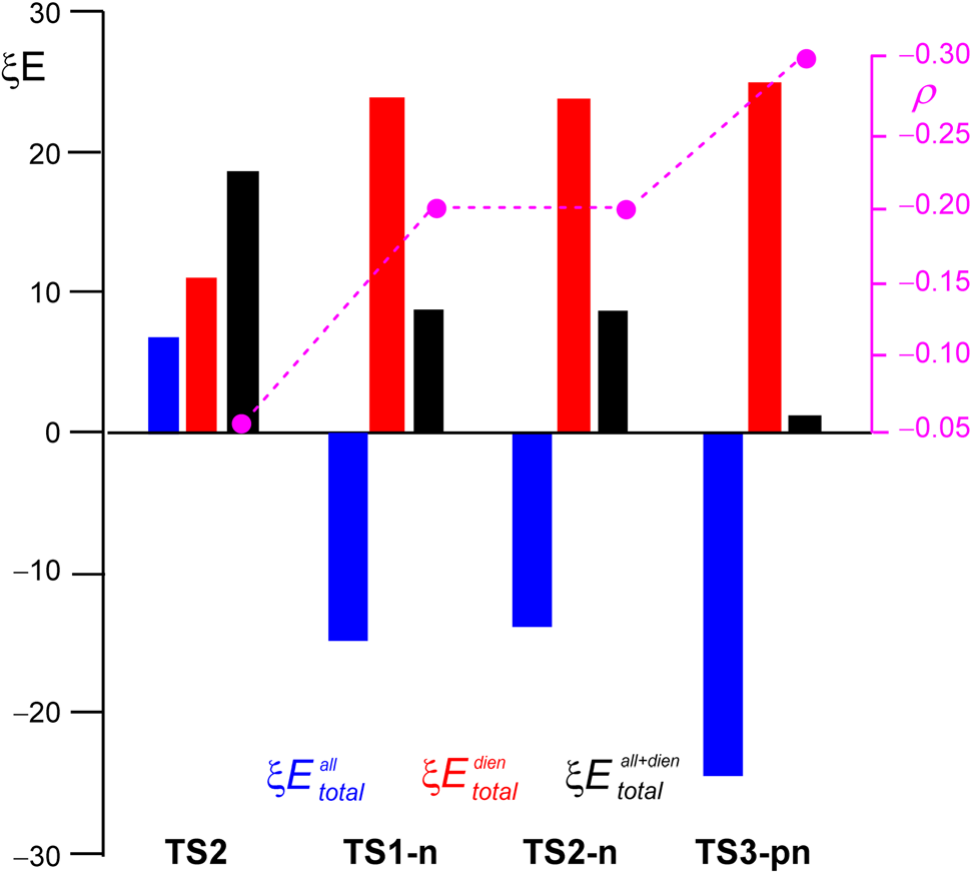
Graphical representation of the sets of the total energies, *ξE*^all^_total_, *ξE*^dien^_total_, and *ξE*^all+dien^_total_, for the TSs given in Table 2. *ξE*^all+dien^_total_ correspond to the RIAE activation energies of these DA reactions. *ξE*^X^_total_ for the allene and diene frameworks are shown in blue and red, respectively, while the black bar represents *ξE*^all+dien^_total_. *ξE*^X^_total_ are reported in kcal mol^−1^. The GEDT values at the TSs, displayed in pink, are expressed as the average number of electrons, e.

Finally, Fig. 10 shows a graphical representation of the total energies (*ξE*^X^_total_) of the allene and diene frameworks at the TSs, shown in red and blue, respectively. The total energies (*ξE*^all+dien^_total_), shown in black, represent the RIAE relative energies of these DA reactions. As can be seen, along this series of DA reactions, the total energies (*ξE*^all^_total_) associated with the allene framework at non-polar TS2 was positive and unfavorable, while those associated with the electrophilic allene framework at polar TS1-n, TS2-n and TS3-pn became negative and favorable. On the other hand, the total energies (*ξE*^dien^_total_) associated with the nucleophilic diene framework became more positive and unfavorable. Overall, these phenomena showed a decrease in the RIAE activation energies with the increase of the polar character of the DA reaction. As shown in Fig. 10, the behaviors of TS1-n and TS2-n are very similar, illustrating similar RIAE relative energies.

The stabilization of the electrophilic allene framework in the three polar DA reactions proportionally increased with the magnitude of the GEDT occurring at TSs (see Fig. 7 and 8). This observation at TS1-n, TS2-n and TS3-pn aligned with Parr's definition of the electrophilicity (*ω*) index, which was defined as *a measure of the energy stabilization of a molecule when it acquires an additional amount of electron density,* Δ*N, from the environment*;^12^ in this polar organic reaction, the nucleophilic species were destabilized.

Fig. 10 shows that the electrophilic sulphonyl allene framework is stabilized at the TSs associated with the polar DA reactions as a consequence of GEDT taking place at the TSs.^23,25,26^ Note that in the non-polar DA reaction, the simplest allene framework was destabilized (see TS2 in Fig. 10). This behavior rejects the “distortion” concept proposed in the distortion/interaction model,^19^ which involves making an energetic comparison between the geometry of the allene framework at the TS and that of the allene reagent at the GS.^30^ As in the distortion/interaction model, the TS geometries were physically divided into the two interacting frameworks, *i.e.* the diene and the ethylene; the ‘distortion energy’ is always positive as a consequence of the loss of the stabilizing effect caused by the GEDT at the TS geometry in polar processes.^23,25,26^ Consequently, the more favorable electrophilic/nucleophilic interactions taking place at the most favorable regioisomeric TS, which are predicted by the analysis of the Parr functions at the GS of the reagents, and not the lower distortion energy as was proposed,^30^ are responsible for the regioselectivity in polar cycloaddition reactions.^64^

## 4. Conclusions

The polar DA reactions of two electrophilic sulphonyl allenes (5 and 17) with two nucleophilic cyclic dienes, Cp (6) and Cp-OMe (18), were studied within the MEDT at the M06-2X/6-311G(d,p) computational level in order to investigate the reactivity and selectivities found experimentally.^8^ ELF of the two sulphonyl allenes showed that the substitution of the methyl group by the experimental phenyl one in the sulphonyl substituent did not modify the electronic structure of the allene. The EW sulphonyl substituents on the C1 carbon of these allenes only slightly polarized the electron density of the C2–C3 double bond towards the C1–C2 one. The analysis of the DFT-based reactivity indices indicated that the sulphonyl allenes (5 and 17) were strong electrophiles, while the cyclic dienes (6 and 18) were strong nucleophiles. Consequently, it was predicted that the corresponding DA reactions would have a high polar character in reactions categorized as FEDF.

The polar DA reaction between the methylsulphonyl allene (17) and Cp (6) presented a low activation enthalpy of 11.3 kcal mol^−1^, which was strongly exothermic with a reaction enthalpy of −39.9 kcal mol^−1^. The DA reaction was kinetically controlled. This polar DA reaction was completely site-selective and exhibited some *endo* stereoselectivity, which was in complete agreement with the experimental outcomes.^8^ The experimental polar DA reaction between phenylsulphonyl allene (5) and Cp (6) presented a low activation enthalpy of 10.4 kcal mol^−1^, which was strongly exothermic by −39.0 kcal mol^−1^. The polar DA reaction between the methylsulphonyl allene (17) and substituted Cp-OMe (18) presented a low activation enthalpy of 4.1 kcal mol^−1^, which was strongly exothermic by −43.7 kcal mol^−1^. This polar DA reaction was completely *para*-regioselective, in complete agreement with the experimental DA reactions involving ER-substituted dienes.^8^

The analysis of the geometries of the more favorable TSs indicated that they had an asynchronous C–C single bond formation, which was controlled by the approach of the nucleophile to the most electrophilic C2 carbon of these allenes, which was in agreement with the previous analysis of the Parr functions. The analysis of the GEDT at the more favorable TSs pointed out the polar character of these DA reactions classified by FEDF. ELF of TSs showed the earlier nature of these non-concerted polar DA reactions. The formation of the new C–C single bonds did not begin at TSs.

Finally, a RIAE analysis of these polar DA reactions established that along this series of reactions, while the *ξE*^all^_total_ energies associated with the sulphonyl allene framework were stabilizing between −13.7 and −24.0 kcal mol^−1^, those associated with the diene framework were destabilizing between 22.5 and 25.0 kcal mol^−1^. The high stabilization of the allene framework with the increase in GEDT accounted for the reduction of the activation energies of these polar DA reactions.

A detailed analysis of the factors responsible for the strong stabilizing intra-atomic energies (*ξE*^all^_intra_) in the polar DA reactions involving these sulphonyl allenes revealed that the sulfur atom of the sulphonyl group was the key contributor. This strong stabilization, resulting from GEDT occurring at polar TSs, was the main atomic-level electronic factor responsible for the reduction of the activation energies.

The presented MEDT studies of the DA reaction between the sulphonyl allenes and nucleophilic cyclic dienes will provide deep insight into the mechanism of the title reactions. They will also allow us to formulate conclusions regarding the synthesis of subsequent reactions involving allenes.

## Author contributions

Conceptualization, A. K. Z. and L. R. D.; methodology, A. K. Z. and L. R. D.; software, A. K. Z. and L. R. D.; validation, A. K. Z. and L. R. D.; formal analysis, A. K. Z. and L. R. D.; investigation, A. K. Z. and L. R. D.; resources, A. K. Z. and L. R. D.; data curation, A. K. Z. and L. R. D.; writing—original draft preparation, A. K. Z. and L. R. D.; writing—review and editing, A. B., D. G., A. K. Z. and L. R. D.; visualization, A. B., D. G., A. K. Z. and L. R. D.; supervision, A. K. Z. and L. R. D.; project administration, A. K. Z. and L. R. D.; and funding acquisition, A. K. Z. and L. R. D. All authors have read and agreed to the published version of the manuscript.

## Conflicts of interest

The authors declare no conflicts of interest.

## Supplementary Material

RA-016-D6RA02126C-s001

## Data Availability

The original contributions presented in this study are included in the article. Further inquiries can be directed to the corresponding author. Supplementary information (SI) is available. See DOI: https://doi.org/10.1039/d6ra02126c.
