# Supplementary material for: Electrophilic allenes participate in polar Diels–Alder reactions: study of the reactivity and site-, regio- and stereoselectivity from a molecular electron density theory perspective
Source: RSC Adv. 2026 Jun 1;16(32):29542–55. doi: 10.1039/d6ra02126c (PMC13227494; doi:10.1039/d6ra02126c)
Supplement: RA-016-D6RA02126C-s001 [file RA-016-D6RA02126C-s001.pdf]

Supplementary Information (SI) for RSC Advances.
This journal is © The Royal Society of Chemistry 2026

Supplementary Material

Electrophilic Allenes Participating in Polar Diels-Alder reactions. Study of the Reactivity and Site, Regio and Stereo Selectivity from A Molecular Electron Density Theory Perspective

Alicja Bigosińska[a], Dominika Gondek[a], Luis R. Domingo[b,*], Agnieszka Kącka-Zych[a,*]

[a] Cracow University of Technology, Faculty of Chemical Engineering and Technology, Department of Organic Chemistry and Technology, Warszawska 24, 31-155, Cracow, Poland;
agnieszka.kacka-zych@pk.edu.pl
[b] Independent Researcher, Av. Tirso de Molina 20, 46015, Valencia, Spain;
luisrdomingo@gmail.com

Index

S2 Scheme with the Diels-Alder reaction of simplest allene **3** with Cp **6**.

S3 Figure with the M06-2x/6-311G(d,p) optimized geometry in toluene of **TS4** involved in the Diels-Alder reaction of the simplest allene **3** with Cp **6**.

S3 Figure with the ELF basin attractor positions, along with the most relevant valence basin populations of **TS4** associated with the Diels-Alder reaction between the simplest allenes **3** and Cp **6**.

S4 Table with the electrophilic P_k^+ and nucleophilic P_k^- and Parr functions of the reagents.

S5 Table with the M06-2x/6-311G(d,p) electronic energies, enthalpies, entropies S, and Gibbs free energies, computed in toluene at 80 ºC, of the stationary points associated with the Diels-Alder reactions of allene **3** and sulphonyl allenes **5** and **17** with the cyclic dienes **6** and **18**.

.

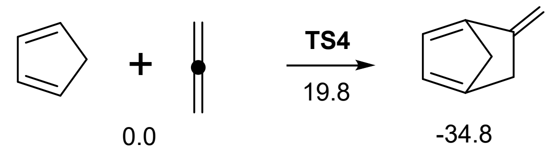

6 **3** **24**

Scheme S1. Diels-Alder reaction of simplest allene **3** with Cp **6**. Relative enthalpies in toluene are given in kcal·mol^{-1}.

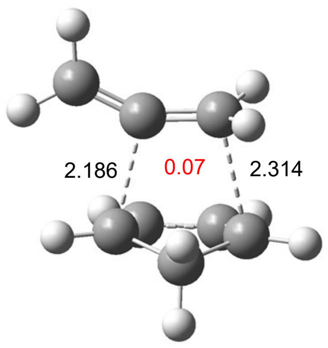

TS4

Figure S1. M06-2x/6-311G(d,p) Optimized geometry in toluene of **TS4** involved in the Diels-Alder reaction of the simplest allene **3** with Cp **6**. The GEDT value, in red, is given in average number of electrons, e.

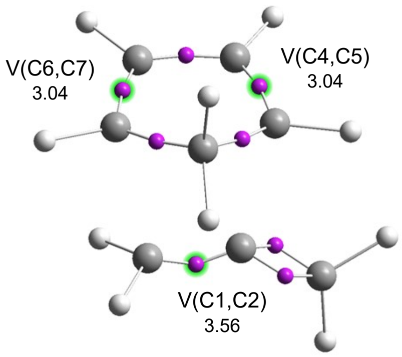

TS2

Figure S2. ELF basin attractor positions, along with the most relevant valence basin populations of **TS4** associated with the Diels-Alder reaction between the simplest allene **3** and Cp **6**. The electron populations are in average number of electrons e.

Table S1. Electrophilic P_k^+ and nucleophilic P_k^- and Parr functions of the reagents.

		**Electrophilic Parr function**	**Nucleophilic Parr function**
		P_k^+	P_k^-
**5**	C_1	0.06	0.21
	C_2	0.31	0.11
	C_3	0.05	0.18
**17**	C_1	0.09	0.31
	C_2	0.60	0.11
	C_3	0.13	0.23
**6**	C_4	-0.09	-0.06
	C_5	0.42	0.50
	C_6	0.07	0.05
	C_7	0.07	0.05
	C_8	0.42	0.50
**7**	C_4	-0.08	-0.06
	C_5	0.51	0.25
	C_6	0.11	-0.03
	C_7	0.06	0.03
	C_8	0.29	0.74
**18**	C_4	0.43	0.48
	C_5	0.03	-0.07
	C_6	0.09	0.32
	C_7	0.43	0.14

Table S2. M06-2x/6-311G(d,p) electronic energies, E in a.u., enthalpies, H in a.u., entropies S in cal·mol^{-1}·K^{-1}, Gibbs free energies, G in a.u., computed in toluene at 80 ºC, of the stationary points associated with the Diels-Alder reactions of allene **3** and sulphonyl allenes **5** and **17** with the cyclic dienes **6** and **18**.

	E	H	S	G
**17**	-704.491020	-704.384321		-704.436447
**6**	-194.058193	-193.958299		-193.997655
**TS1-n**	-898.533921	-898.325502	10.7	-898.390227
**TS1-x**	-898.532958	-898.324586	11.3	-898.389757
**TS-23**	-898.527337	-898.319190	14.7	-898.386928
**19**	-898.615991	-898.403066	-37.9	-898.465595
**20**	-898.619281	-898.406145	-39.9	-898.467488
**21**	-898.618328	-898.405244	-39.3	-898.467785
**5**	-896.194118	-896.029454		-896.093142
**TS2-n**	-1090.237447	-1089.971153	10.4	-1090.047533
**8**	-1090.320955	-1090.049974	-39.0	-1090.122501
**18**	-308.566512	-308.430107		-308.478340
**TS3-pn**	-1013.052903	-1012.807969	4.1	-1012.880831
**TS3-mn**	-1013.046670	-1012.801701	8.0	-1012.873051
**22**	-1013.133870	-1012.884143	-43.7	-1012.953615
**23**	-1013.134119	-1012.884367	-43.9	-1012.952540
**3**	-116.62758	-116.566116		-116.601626
**TS-4**	-310.656007	-310.492846	19.8	-310.542441
**24**	-310.747872	-310.579929	-34.8	-310.626674
